# Update on Limbal Stem Cell Transplantation

**DOI:** 10.4103/0974-9233.61211

**Published:** 2010

**Authors:** Pejman Bakhtiari, Ali Djalilian

**Affiliations:** University of Illinois Eye and Ear Infirmary, Chicago, USA

**Keywords:** Allogenic Transplantation, Autologous Transplantation, Limbal Stem Cell, Review, Stem Cell Deficiency, Transplantation

## Abstract

Limbal epithelial stem cells are the primary source of corneal epithelial cell regeneration. Limbal stem cell deficiency (LSCD) can develop in traumatic, immunologic, or genetic diseases that affect the ocular surface. LSCD leads to conjunctivalization, with corneal vascularization and opacification and subsequent loss of vision. Limbal stem cell transplantation is a surgical treatment to address LSCD and restore a corneal epithelial phenotype. Based on the source of cells, limbal transplant can be autologous or allogenic. Many surgical techniques are defined according to the source of the stem cells and the carrier tissues that are used. More recently, *ex vivo* expanded bioengineered epithelial cells have been used to reconstruct the corneal surface using autologous cells to eliminate the risk of rejection. Before transplantation, a systematic exam of the lids, eyelashes, fornices, and aqueous tears is mandatory and every effort should be made to optimize ocular surface health and control inflammation to enhance the chances of graft survival. Postoperative care is also another major determinant of success. Any factor that destabilizes the ocular surface needs to be addressed. In addition, systemic and topical immunosuppressants are also needed in all allograft recipients. In addition to pre-operative and postoperative care and the surgery itself, the etiology of LSCD also has an impact on the outcome. The prognosis of inflammatory diseases such as Stevens-Johnson syndrome is the worst among disorders causing LSCD.

## INTRODUCTION

The healthy cornea is covered by stratified, nonkeratinized epithelium whose integrity is essential for the optical clarity of the cornea.^1^ One of the most important properties of the corneal epithelium is its anti-angiogenic properties. The avascularity of the cornea is highly dependent on the integrity of the corneal epithelium^2^ and also in part on the soluble vascular endothelial growth factor receptor.^3^,^4^ It is well known that the corneal epithelium is renewed and repopulated by a cell population residing in the limbus, known as limbal stem cells.^5^–^7^ One of the important new concepts in limbal stem cell biology is the importance of the limbal “niche.” Stem cell niche is a special microenviroment consisting of several cellular and extracellular components in the vicinity. The niche is responsible for the biologic regulation of stem cells.^8^

## LIMBAL STEM CELL DEFICIENCY

Many diseases can cause LSCD. Hereditary or acquired disease and trauma may cause destructive loss of limbal stem cells, such chemical burns and Stevens-Johnson syndrome. The limbal cell niche may be altered due to conditions such as aniridia.^9^ Table 1 summarizes the diseases and conditions that can cause LSCD. Depending on the extent of limbal involvement, LSCD may be partial or total.

**Table 1:** Corneal diseases manifesting limbal stem cell deficiency

Hereditary
Anirida
Keratitis associated with multiple endocrine deficiency
Epidermal dysplasia (ectrodactyly-ectodermal dysplasia-clefting syndrome, KID syndrome)
Acquired
Chemical or thermal burns
Stevens-Johnson syndrome, toxic epidermal necrolysis
Multiple surgeries or cryotherapies to limbus
Contact lens-induced keratopathy
Severe microbial infection extending to limbus
Antimetabolite uses (5-FU, mitomycin C)
Radiation
Chronic limbitis (vernal, atopy, phlyctenular)
Mucous membrane pemphigoid

Clinical symptoms of LSCD may include photophobia, blurred or decreased vision, tearing or recurrent episode of pain from epithelial breakdown, and history of chronic inflammation and redness.^10^ The clinical signs of LSCD vary depending on its severity, and include:

- loss of limbal anatomy
- irregular, thin epithelium
- stippled fluorescein staining of the area covered by abnormal epithelium
- filaments and erosions
- superficial and deep vascularization
- persistent epithelial defects leading to ulceration, melting, and perforation
- fibrovascular pannus
- scarring, keratinization, and calcification

**Figure 1:**
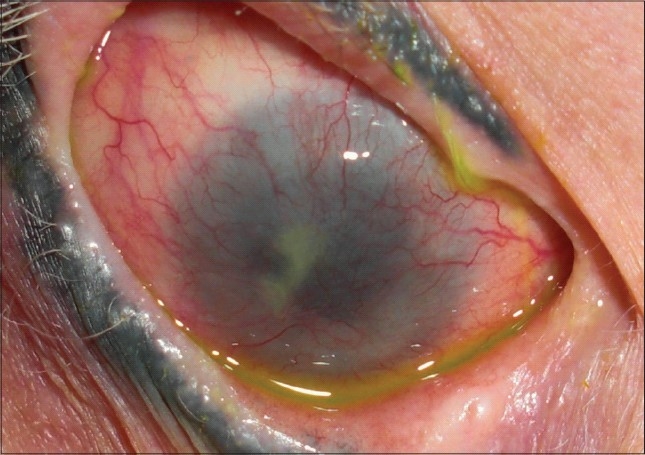
Total stem cell deficiency due to Stevens-Johnson syndrome. Note, corneal conjunctivalization and vessel invasion all around 360°

Figure 1 demonstrates total LSCD in a patient with Stevens-Johnson syndrome.

## LIMBAL STEM CELL TRANSPLANTATION

The goal of treatment for severe LSCD is to re-establish the anatomic and physiologic environment of the ocular surface by reconstruction of the corneal and conjunctival epithelium.^11^

### Importance of pre-operative management

The main objective before transplanting limbal stem cells is to prepare their new “home” and to provide the best opportunity for graft survival. In particular, survival of limbal stem cells depends in part on the limbal niche that is influenced by tear film and vascularity and innervation at the limbus.^12^ Several issues need to be addressed before stem cell transplantation, including optimizing lids and the tear film, controlling inflammation, and the management of glaucoma.

Patients with ocular surface disease often have multiple factors affecting the surface, and a mild abnormality that may be tolerated in a normal eye can compromise the outcome of surgery. Hence, a low threshold to treat adnexal abnormalities before stem cell transplantation is recommended. A pre-operative systematic assessment of the adnexa, including tear film condition, eyelid position, lagophthalmos, and fornix depth is mandatory.^13^–^15^ Overall, the health and function of eyelids, fornices, and tear film should be optimized before stem cell grafting to ensure the best chance of epithelial healing.^16^ In cases of severe conjunctival disease and symblepharon, a source of goblet cells is required for conjunctival surface and fornix reconstruction. A variety of donor sites are available for autologous mucous membrane transplantation to the ocular and eyelid surface, including buccal, labial, hard palate, nasal turbinate, and septal mucosa.^17^

Ocular surface inflammation should be suppressed pre-operatively as much as possible. The eye needs to be quiet for at least 3 months before surgery to enhance the chances of survival for transplanting stem cells. We therefore recommend topical and systemic immunosuppression several months prior to stem cell transplantation in patients with significant underlying inflammatory disease, such as atopic disease or Stevens-Johnson syndrome.^18^

The presence and severity of glaucoma can have a significant impact on the outcome. A rise in intraocular pressure is often seen following limbal transplantation, which may be attributed to the use of steroids. Additionally, multiple topical medications are toxic to the transplanted epithelial surface. Hence, there is a lower threshold in managing glaucoma in such patients. We recommend the early placement of a tube shunt in patients on more than one topical medication.^18^

### Surgical techniques for limbal transplantation

Numerous techniques to replace limbal stem cells have been described^19^–^21^ with the common goal of ocular surface restoration. Holland and Schwartz have published nomenclature and classification for ocular surface procedures.^22^ The nomenclature is based on the source of the donor tissue, the carrier tissue employed, and whether conjunctival or limbal tissue is transplanted.^18^ Currently, the main clinical procedures that are performed include a conjunctival limbal autograft (CLAU) using tissue from the fellow eye; a living related conjunctival limbal allograft (lr-CLAL), where a living relative donates conjunctiva and limbal tissue; and keratolimbal allograft (KLAL), utilizing a cadaveric donor where the peripheral cornea is used to transfer the limbal stem cells.^18^ More recently, *ex vivo* expanded limbal stem cells or oral mucosa cells have also been used successfully to reconstruct the ocular surface.^17^,^23^,^24^ The latest reports and variations on these procedures are described below.

### Conjunctival limbal autograft

In unilateral LSCD, the healthy fellow eye is the most suitable source of limbal stem cell. Kenyon and Tseng in 1989 were the first to report results of a large series of CLAU transplantation on 21 patients. This technique was actually a modification of Thoft's conjunctival transplant procedure on extending the grafts of bulbar conjunctiva 0.5 mm onto the clear cornea to obtain limbal stem cells.^25^ Harvesting begins in the conjunctiva, including 4-5 mm of conjunctival tissue, moving anteriorly to remove a partial-thickness limbal epithelium of about one-third thickness. Preparation of the recipient eye begins with 360° peritomy and sharp and blunt dissection of the fibrovascular pannus over the cornea and securing the transplanting block at the 6 and 12 o’ clock positions.^18^ We prefer to use two blocks of tissue, each 2’ clock hours in circumferential extension. Recent update on the technique is using fibrin glue instead of sutures to secure the transplant.

The main concern with this procedure is inducing stem cell deficiency in the fellow eye. No complications in the fellow eyes were reported in Kenyon and Tseng's series and the risk to the donor eye appears extremely low if the donor eye is truly healthy with no long-term contact lens usage or subclinical exposure to original trauma and less than 6’ clock hours of limbal tissue is removed.^25^ Clearly, CLAU is not an option for patients with bilateral disease.

Although CLAU is an autograft and there is no risk of immunologic rejection, like all forms of stem cell transplantations, it must be considered only after adequate control of ocular inflammation to provide a better environment for transplanting cells.

### Living related conjunctival limbal allograft (lr CLAL)

The lr-CLAL technique is similar to CLAU. However, the source of stem cell is a living relative instead of the fellow eye.

HLA typing on all potential donors is helpful in finding more compatible tissue to transplant.

Potential donors with long-term contact lens usage and glaucoma, who may eventually require trabeculectomy, should be excluded.^26^ Serologic testing of potential donors for syphillis, hepatitis B and C, and human immunodeficiency virus infection should be performed to avoid risk of transmission to the recipient.

This procedure provides conjunctival and limbal stem cells to the host with some degree of histocompatiblity. As discussed above, damage to the donor's eye is very unlikely, but should be considered. In addition, the risk of rejection exists and patients require systemic immunosuppression therapy.

Lr-CLAL provides healthy conjunctival tissue in addition to stem cells, but it does not cover 360° of the limbus and leaves gaps in areas that may allow conjunctivalization of the surface in total stem cell deficiency. Hence, lr-CLAL may result in better outcome for patients with partial stem cell deficiency. In the most severe cases of ocular surface disease, if the patient has extensive conjuctival disease and total LSCD, combined KLAL and lr-CLAL, called the “Cincinnati procedure,” maximizes the advantages inherent in both procedures.^18^ Introduced by Holland *et al*., the Cincinnati procedure begins with recipient eye preparation as for standard KLAL.^18^ Conjunctival tissue is placed superiorly and inferiorly and keratolimbal tissue is used to fill in the gaps nasally and temporally.^18^ Systemic immunosuppression is required and these patients may be at higher risk for immunologic rejection because two different types of antigenic tissues are used.^27^

### Keratolimbal allograft

KLAL uses peripheral cornea as the carrier for allogenic cadaveric stem cells. In 1984, Thoft introduced keratoepithelioplasty by harvesting a rim of cadaveric cornea and transplanting it to a recipient after total superficial keratectomy.^26^,^28^ In 1994, Tsai and Tseng used a whole globe and they harvested an annular ring of limbal tissue and termed the procedure human allograft limbal transplantation.^29^

In 1995, Tsubota *et al*. used stored corneoscleral rim for limbal stem cell transplantation and termed their procedure limbal allograft transplantation.^30^ In 1996, Holland *et al*. modified Tsubota's technique using two stored corneoscleral rims. In this procedure, the central cornea is removed with a 7.50-mm trephine.^19^ The rim is bisected and excess peripheral tissue is removed. Then, lamellar dissection to remove the posterior two-thirds of the stroma along with Descemet's membrane and endothelium is performed. The host eye surface is prepared by performing 360° conjunctival peritomy and releasing areas of symblepharon. Superficial keratectomy is performed to peel off pannus and conjunctivalized tissue, creating as smooth a surface as possible. Amniotic membrane can be transplanted at this time. Amniotic membrane has been shown to reduce inflammation and scarring and facilitate epithelial wave movement.^31^

**Figure 2:**
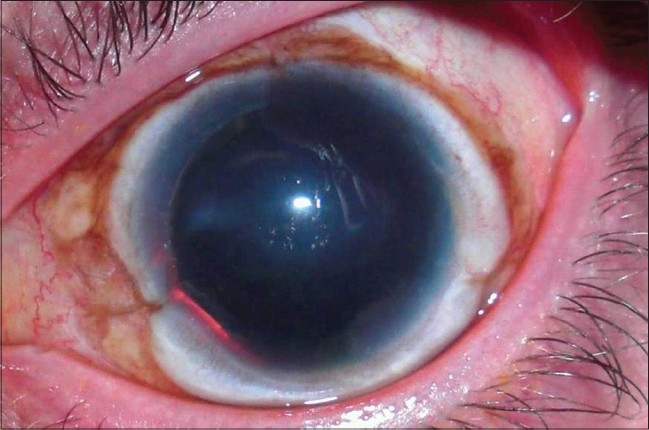
Patient with aniridia 6 months after keratolimbal allograft

The prepared limbal graf ts are secured to the eye using 10-0 nylon sutures, trying to match donor's and recipient's limbus.^26^,^32^ More recently, fibrin glue was employed to secure KLAL blocks in place. This will add intra- and postoperative patient comfort and may result in a smoother ocular surface postoperatively (presented at Eye Bank Association of American Meeting, San Francisco, 2009). Figure 2 demonstrates the postoperative appearance of a patient 6 months after KLAL using fibrin glue.

KLAL does not provide conjunctival tissue and therefore it is the procedure of choice for patients with primary limbal involvement with minimal conjunctival involvement, such as aniridia. Patients with total LSCD and conjunctival involvement may benefit more from lr-CLAL combined with KLAL.^26^

Kim *et al*. reported 89 patients with a follow-up of 4.70 years. Kim *et al*. found that 73% of the patients had stable ocular surface at the last follow-up and subsequent keratoplasties were successful in 65% of the patients.^26^ Nearly all of the patients received triple immunosuppressive therapy, initial patients with oral prednisone, cyclosporine, and azathioprine and, more recently, with prednisone, tacrolimus, and mycofenolate mofetil.^26^

### *Ex vivo* stem cell expansion

Transplantation of cultivated limbal stem cells for the treatment of partial and total LSCD has been recently developed. Pelligrini *et al*. reported their result of transplanting *ex vivo* expanded autologous limbal stem cells from the fellow eyes of patients with unilateral alkaline injury.^33^ They reported successful corneal epithelialization and stability of regenerated epithelium up to 2 years after treatment, with improvement in patient discomfort and visual acuity. In 2000, Tsai *et al*. reported results of transplantation of autologous cultivated stem cells in six patients, five with partial and one with total stem cell deficiency from chemical burns.^34^ They used amniotic membrane as the carrier tissue.^34^

*Ex vivo* cultivated stem cell transplantation provides a useful method to restore the stem cell population. Obtaining autologous tissue in unilateral involvement eliminates the need for immunosuppression. Additionally, removing a small limbal biopsy of about 1-2 mm from the healthy eye does not pose a considerable risk. However, longer-term studies are required to determine the long-term efficacy of the procedure. In particular, the limbal niche may need to be restored in order for the procedure to be effective over time.

Ang *et al*. reported the use of autologous serum derived from cultivated oral epithelial transplant for the treatment of severe ocular surface disease with total LSCD.^35^ This method uses completely autologous xenobiotic-free bioengineered ocular equivalent for clinical transplantation.^18^,^35^,^36^ All eyes achieved complete corneal epithelialization within 2-5 days and remained stable after a mean follow-up of 12 months.^35^

### Postoperative management and Immunosuppression

Postoperative management of patients who undergo limbal stem cell transplantation is one of the most important factors that determine the success rate and outcome. Postoperatively, topical antibiotic is used until the surface is completely epithelialized. Topical steroids are used to reduce inflammation and topical cyclosporine or tacrolimus may be added to the regimen as required.^37^ The health of the ocular surface should be optimized with the use of nonpreserved artificial tears, punctal occlusion, bandage lens, tarsorrhaphy, and trichiasis removal. Any factor that destabilizes the ocular surface needs to be addressed aggressively and quickly.

Transplantation of an allograft poses the risk of rejection even in HLA-matched recipients. Therefore, all allografts such as KLAL and lr-CLAL need prolonged systemic immunosuppression, which could span their lifetime.^38^ The goal of immunosuppression is to eliminate eye inflammation and prevent allograft rejection. Topical immunosuppressants are usually insufficient in controlling allograft rejection after KLAL. In a series by Kim *et al*., the success rate after KLAL was 87% in patients receiving systemic immunosuppression *vs*. 29% in patients treated with only topical immunosuppression.^26^

We prefer combined immunosuppressive therapy, including steroids, tacrolimus, and mycophenolate mofetil, as summarized in Table 2. Combined systemic immunosuppression based on mycophenolate mofetil and tacrolimus seems to be more effective and safer than cyclosporine A alone.^39^ Tseng *et al*. also showed the role of combined immunosuppression with tacrolimus and mycophenolate mofetil on long-term maintenance of functional graft.^16^ Alloway *et al*. reported that KLAL patients on mycofenolate mofetil and tacrolimus had significantly fewer adverse systemic events compared to age-matched renal transplant patients.^40^ In general, it is recommended to co-manage the patient with an organ transplant immunologist to minimize the risk of adverse effects.

**Table 2:** Immunosuppressive regimen after limbal stem cell allograft transplantation

Medication	Dosage and Duration
Corticosteroids	
Topical	Qd-qid, indefinitely
Oral	0.5-1 mg/kg/d, taper
Over 3-4 months	
Cyclosporin A	
Topical	0.05% qid, indefinitely
Oral	3 mg/kg/d, 12-18 months
OR	
Tacrolimus	3-4 mg q12h, 12-18 months
Azathioprine	100 mg/d, 18-24 months
OR	
Mycophenolate	1,000 mg bid, 18-24 months

### Prognosis and outcome

Outcome of stem cell transplantation can be adversely affected by several risk factors threatening ocular surface health.^38^ Ocular surface health depends on a stable tear film, which occurs due to the adnexal glands, eyelids, neuroanatomic integration of two neural reflexes controlling the secretion of different tear components, and eyelid closure.^16^,^41^

There are several risk factors for transplanted stem cell survival. Liang *et al*. identified pre-operative clinical characteristics and risk factors that lead to ocular surface deficits, which included: Infrequent blinking, blink-related microtrauma, conjunctival inflammation, increased intraocular pressure, aqueous-deficient dry eye, and previous failed corneal or stem cell graft.^16^ Holland showed keratinization of the conjunctiva as a risk factor for KLAL failure.^42^

Current consensus is that autologous limbal grafts (CLAU) have a better prognosis than allogenic grafts (KLAL).^43^ The most significant advantage of CLAU is the absence of immunologic rejection. However, persistent inflammation of the ocular surface resulting from the original disease, infection, or abnormal eyelids also can cause loss of donor limbal tissue.^43^ Although KLAL rejection is considered the major cause of failure, other risk factors, such as keratinization,^44^ symblepharon,^45^ inflammation,^46^ and dry eye,^47^ have been implicated.

Solomon *et al*. reported that patients with Stevens-Johnson syndrome have the worst prognosis after KLAL in terms of ambulatory vision and success of penetrating keratoplasty. They also found younger patient age and performing penetrating keratoplasty simultaneously with KLAL resulted in poor prognosis and poor ambulatory vision.^48^ Solomon *et al*. found that simultaneous PKP with KLAL decreases the KLAL survival, although the difference was not statistically significant (81% *vs*. 59%).^48^ Their data did not demonstrate numbers of previous procedures, previous glaucoma, or lid surgery as a prognostic factor of KLAL survival.^48^ Solomon *et al*. also found that the success of penetrating keratoplasty decreased in eyes undergoing simultaneous surgeries. Shimatzki *et al*. reported endothelial rejection followed by decompensation in 10 of the 16 eyes that underwent PKP and KLAL in the same session.^49^

## CONCLUSION

To achieve the best possible success rates in limbal transplantation, the following points are imperative:

1. Pre-operative correction of eyelid, eyelashes, and fornix abnormalities prior to transplantation in order to optimize tear film status
2. Adequately control inflammation using topical and systemic medications for at least 3-6 months before surgery
3. Management of glaucoma with a lower threshold to surgical inter vention before limbal transplantation
4. Choosing the best method to restore limbal stem cells:

CLAU for unilateral disease

- KL AL for bilateral limbal deficiency with minimal to moderate conjunctival disease.
- Lr-CLAL for bilateral limbal deficiency (preferably partial limbal involvement) with moderate to severe conjunctival disease.
- Combined lr-CLAL and KLAL for bilateral limbal deficiency and severe conjunctival disease.
